# Platelets enhance malignant behaviours of gastric cancer cells via direct contacts

**DOI:** 10.1038/s41416-020-01134-7

**Published:** 2020-10-28

**Authors:** Ryo Saito, Katsutoshi Shoda, Suguru Maruyama, Atsushi Yamamoto, Koichi Takiguchi, Shinji Furuya, Naohiro Hosomura, Hidenori Akaike, Yoshihiko Kawaguchi, Hidetake Amemiya, Hiromichi Kawaida, Makoto Sudo, Shingo Inoue, Hiroshi Kono, Katsue Suzuki-Inoue, Daisuke Ichikawa

**Affiliations:** 1 First Department of Surgery, Faculty of Medicine, University of Yamanashi, Chuo, Japan; 2 Department of Clinical and Laboratory, Faculty of Medicine, University of Yamanashi, Chuo, Japan

**Keywords:** Gastric cancer, Cancer microenvironment

## Abstract

In this study, we aimed to analyse human cancer cell–platelet interactions in functional cell analyses and explore the molecular mechanisms behind tumour progression. Various functional analyses of gastric cancer (GC) cells were performed after direct/indirect co-incubation with platelets derived from GC patients. Further detailed expression and signalling analyses were performed after co-culture with direct and indirect GC cells–platelet contact. Malignant behaviours of cancer cells, such as proliferation, migration, invasion and adhesion, were significantly enhanced after direct co-incubation with platelets. Microarray analyses demonstrated changes in multiple genes, including epithelial–mesenchymal transition (EMT)-related genes. Among them, matrix metalloproteinase 9 was notably upregulated, which was validated by quantitative reverse transcription–polymerase chain reaction and western blot. Further, this change was only observed after direct co-incubation with platelets. This study demonstrated that platelets from GC patients promote malignant behaviours of GC cells through EMT-related signalling, especially by direct contact with tumour cells.

## Background

Recent studies have elucidated that interactions of tumour cells with the surrounding tissues play pivotal roles in tumour invasion and progression.^1^ Other than cancer-associated fibroblasts, immune cells, one of blood cells, are also considered to be deeply involved in tumour progression.^2,3^ However, there are only a few studies about the interactions of cancer cells with platelets, even in the same blood cells. Although previous experiments using laboratory animals have demonstrated that platelets play various roles in cancer, including hematogenous metastasis, the detailed mechanisms by which platelets play pro-tumorigenic roles still remain unclear.^4,5^

Our previous clinical studies indicated that intraoperative haemorrhage is more likely to develop peritoneal recurrence.^6^ These findings prompted us to investigate the impact of platelets on tumour malignancy, especially concerning the development of peritoneal metastasis, by basic and clinical studies using human samples. In this study, we aimed to analyse human cancer cell–platelet interactions in functional cell analyses and explore the molecular mechanisms behind tumour progression.

## Methods

### Human platelet samples and cell lines

Blood samples were collected from patients with advanced GC, before therapeutic interventions. Patient characteristics are shown in Supplementary Table 1. Platelets were purified from whole- blood samples. The human GC cell lines NUGC-3 and MKN74, and the human normal mesothelial cell line Met-5A, were used in analyses of cell functions and molecular mechanisms.

### Observation of cancer cell–platelet complex and cell functional analyses

NUGC-3 co-cultured with platelets was observed by scanning electron microscopy (SEM). The functions of GC cells co-cultured with platelets were evaluated for proliferation, migration and invasion as well as adhesion abilities to mesothelial cells, each compared to those of GC cells not co-cultured with platelets. For comparison, these functional analyses were carried out in conditions of both direct and indirect contact between GC cells and platelets, using novel experimental systems (Supplementary Fig. 1).

### Molecular mechanism analyses

Molecular analyses with a microarray were performed using NUGC-3 cells co-cultured with platelets for 48 h compared to those cultured without platelets as a control. Microarray analyses were also performed in GC cells co-cultured with platelets for 15 min and in GC cells co-cultured with platelets separated by 0.4-µm pore membranes. The former condition was helpful to eliminate the gene expression changes affected by intra-platelet mRNA, and the latter represented gene expression changes affected only by molecules and exosomes secreted by platelets (Supplementary Fig. 2).

Following microarray analysis, enrichment analyses of the altered mRNAs and functional pathways were performed using GO enrichment and KEGG pathway analysis, respectively. Then, validation analysis of the mRNA expression in each condition of cancer–platelet contact was performed using quantitative reverse transcription–polymerase chain reaction. Further, protein expression was also validated by western blotting. *P* < 0.05 was considered to indicate a statistically significant result in all analyses in this study. For detailed methods of each experiment, see Supplementary files.

## Results

### Observation of electron microscopy and cell functional analyses

**Fig. 1:**
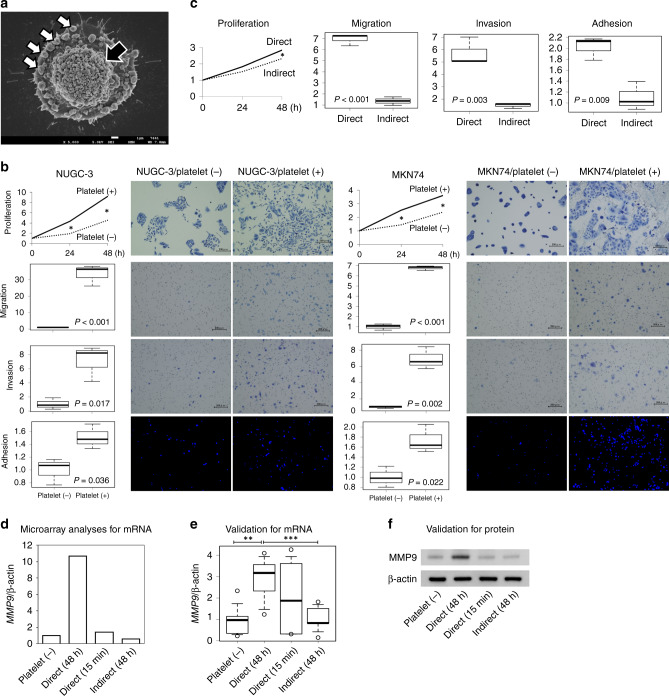
Analyses of cell functions and molecular mechanisms derived by co-incubation with platelets. **a** SEM imaging for cancer cell–platelet contacts. Complexes were composed of a number of platelets (white arrows) surrounding GC cells (a black arrow). **b** Functional analyses. Top: proliferation assay. Significant differences were indicated with an asterisk (*P < 0.05). Second top: migration assay. Third top: invasion assay. Bottom: adhesion assay to mesothelial cells. Only GC cells were pre-labelled and observed as blue points by fluorescence microscope, in contrast to pre-cultured Met-5a. **c** Comparison between the effects of direct and indirect contact with platelets on malignant behaviour of GC cells. Enhanced malignant behaviour of GC cells was observed upon direct contact with platelets and not upon indirect contact. **d** *MMP9* mRNA expression levels in microarray analyses. **e** Validation experiment for *MMP9 mRNA* expression. *MMP9 mRNA* level was significantly upregulated after direct contacts for 48 h, compared to control (**P = 0.004) and indirect contact (***P = 0.006). **f** Western blotting. An increased MMP9 by direct contact with platelets was confirmed even in protein level. GC gastric cancer, MMP9 matrix metalloproteinase 9.

SEM imaging demonstrated that GC cells were surrounded by a number of platelets and adhered to the platelets in co-culture in as little as 15 min (Fig. 1a). The results of functional analyses are shown in Fig. 1b. The proliferation ability of GC cells was significantly enhanced by co-culture with platelets at all the time points evaluated (24 h and 48 h) in both GC cell lines, although the difference was more marked in NUGC-3 cells (*P* < 0.001, *P* = 0.001) than in MKN74 cells (*P* = 0.014, *P* = 0.018). The migration assay revealed that platelets significantly improved the migration abilities of both GC cell lines (*P* < 0.001 for both). Similarly, the invasion assay also revealed significantly more invasion after co-culture with platelets in both cell lines (NUGC-3, *P* = 0.017 and MKN74, *P* = 0.002). Adhesion assays using mesothelial Met-5A cells revealed that co-culture with platelets significantly increased the adhesion of GC cells (NUGC-3, *P* = 0.036 and MKN74, *P* = 0.022). Further, these enhanced malignant behaviours were observed after direct contact of GC cells with platelets, but not after indirect contact, in both NUGC-3 (Fig. 1c) and MKN74 (data not shown) cell lines.

### Microarray and enrichment analyses

The microarray analyses identified 617 upregulated and 734 downregulated mRNAs with more than 1.5-fold differences in GC cells co-cultured with platelets for 48 h (Supplementary Table 2). The results of further GO and KEGG pathway analyses are shown in Supplementary Tables 3 and 4. The GO analysis indicated significant changes in genes involved in cell proliferation (*P* < 0.001), cell adhesion (*P* = 0.003) and cell migration (*P* = 0.003).

Next, alterations in genes related to the epithelial–mesenchymal transition (EMT), which is related to the migration and invasion abilities of cancer cells, were assessed (Supplementary Table 5). There were alterations in several EMT-related mRNAs in GC cells co-cultured with platelets, such as upregulation of matrix metallopeptidase 9 (*MMP9)* and integrin subunit alpha 5, and downregulation of claudin 1 and cadherin 1 (Supplementary Fig. 3). *MMP9*, which showed the most remarkable change in the microarray analyses, was used in further studies.

### Validation in various contact models with platelets

The results of microarray analyses in various contact models clearly demonstrated that the *MMP9 mRNA* expression was extremely high only in direct contact for a 48-h model (Fig. 1d). Meanwhile, the *MMP9* level was extremely low in both direct contact for 15 min and indirect contact for 48 h, indicating that the upregulation in *MMP9* resulted from direct adhesion between GC cells and platelets. The results were also confirmed in validation experiments for mRNA expression in multiple samples (Fig. 1e), and for protein expression (Fig. 1f).

## Discussion

The results of this study clearly demonstrated that platelets enhanced several malignant behaviours of GC cells. The functional changes in migration and invasion were the most remarkable changes. All of these malignant changes have advantages for the development of peritoneal metastasis. Further detailed molecular analyses indicated that cancer cell–platelet interaction induced the expression of EMT-related genes in GC cells, and *MMP9* was the most notably changed gene in the microarray analyses. One possible mechanism for these malignant changes is cellular stimulation by the adherence of platelets to cancer cells. The other is the influence of protein and exosomes secreted from platelets.^7^ Our further detailed co-culture analyses clearly demonstrated that enhanced malignant behaviours and significant elevation of *MMP9* expression was observed only in conditions of direct cancer cell–platelet contact but not in transmembrane co-cultured condition. These results indicate that only direct adherence of platelets to cancer cells enhances malignant behaviour due to cancer cell–platelet interaction. Further, in these processes, platelet activation and production of platelet-derived soluble factors might be induced by direct contact with cancer cells.

Previous studies have reported that intraoperative blood loss (IBL) is correlated with prognosis in patients with GC who undergo surgery.^8,9^ Our previous analysis demonstrated that advanced GC patients who experience a large amount of IBL are more likely to develop peritoneal metastases.^6^ Kamei et al. also reported that large IBL during curative gastrectomy for advanced GC is associated with the development of peritoneal recurrence.^10^ These findings can potentially be explained by our current results. There were some limitations in this study, such as the small number of patients. Despite these limitations, our results have the absolute potential to lead to the establishment of a novel concept of cancer progression and metastasis and the development of treatments, based on cancer cell–platelet direct interactions.

In conclusion, this study obviously demonstrated that platelets from GC patients promoted malignant behaviour of GC cells through EMT-related manners, especially by direct contacts.

## Supplementary information

Supplementary material

## Data Availability

The data sets used and analysed in this study are collected at the University of Yamanashi, and they are available from the corresponding author on reasonable request. Most of them are included in this paper.
