# Supplementary material for: Platelets enhance malignant behaviours of gastric cancer cells via direct contacts
Source: Br J Cancer. 2020 Oct 28;124(3):570–3. doi: 10.1038/s41416-020-01134-7 (PMC7851124; doi:10.1038/s41416-020-01134-7)
Supplement: Supplementary file 1 — Supplementary material [file 41416_2020_1134_MOESM1_ESM.docx]

Supplementary files are composed of supplementary Tables, Figures & Figure legends, and a description of Detailed methods.

**Supplementary Table 1. Patients characteristics.**

| **Patients** | **Gender** | **Age** | **Pathological type** | **T** | **N** | **M** | **Stage** |
| --- | --- | --- | --- | --- | --- | --- | --- |
| 1 | M | 84 | undifferentiated | 3 | 0 | 0 | IIA |
| 2 | M | 69 | undifferentiated | 3 | 2 | 0 | IIIA |
| 3 | M | 86 | differentiated | 3 | 1 | 0 | IIB |
| 4 | M | 69 | undifferentiated | 4a | 3b | 0 | IIIC |
| 5 | F | 76 | undifferentiated | 4a | 0 | 0 | IIB |
| 6 | F | 80 | differentiated | 2 | 1 | 0 | IIA |
| 7 | F | 69 | undifferentiated | 4a | 3b | 1 | IV |
| 8 | M | 74 | differentiated | 3 | 1 | 0 | IIB |
| 9 | M | 80 | undifferentiated | 4b | 2 | 0 | IIIB |
| 10 | M | 55 | undifferentiated | 2 | 2 | 0 | IIB |

M; male, F; female.

**Supplementary Table 2. mRNA expression changes after co-incubation with platelets (Top 20).**

| **mRNA** | | **rate to β-actin** | | **rate to control** |
| --- | --- | --- | --- | --- |
| **Gene symbol** | **description** | **Platelet (+)** | **Control** |  |
| ***HBD*** | hemoglobin subunit delta | 0.439 | 0.0059 | 73.8244 |
| ***IFI27*** | interferon, alpha-inducible protein 27 | 0.0213 | 0.0006 | 38.0101 |
| ***MX2*** | MX dynamin like GTPase 2 | 0.1014 | 0.0045 | 22.5232 |
| ***IFI44*** | interferon induced protein 44 | 0.023 | 0.0011 | 20.0109 |
| ***HSD11B1*** | hydroxysteroid (11-beta) dehydrogenase 1 | 0.1875 | 0.0105 | 17.8487 |
| ***OASL*** | 2'-5'-oligoadenylate synthetase-like | 0.0097 | 0.0006 | 17.0829 |
| ***TGFBI*** | transforming growth factor beta induced | 0.0346 | 0.002 | 17.0159 |
| ***IFI6*** | interferon, alpha-inducible protein 6 | 0.648 | 0.0385 | 16.8334 |
| ***IFIT1*** | interferon induced protein with tetratricopeptide repeats 1 | 0.2034 | 0.0121 | 16.8057 |
| ***C4orf26*** | chromosome 4 open reading frame 26 | 0.082 | 0.0052 | 15.8206 |
| ***MX1*** | MX dynamin like GTPase 1 | 0.0913 | 0.0058 | 15.7974 |
| ***CLDN14*** | claudin 14 | 0.0054 | 0.0004 | 15.5065 |
| ***SERPINE1*** | serpin peptidase inhibitor, clade E (nexin, plasminogen activator inhibitor type 1), member 1 | 0.0324 | 0.0022 | 14.71 |
| ***AMTN*** | amelotin | 0.0261 | 0.0018 | 14.2302 |
| ***SAMD9*** | sterile alpha motif domain containing 9 | 0.0354 | 0.0026 | 13.8533 |
| ***MX2*** | MX dynamin like GTPase 2 | 0.0437 | 0.0032 | 13.6813 |
| ***STEAP1*** | six transmembrane epithelial antigen of the prostate 1 | 0.0076 | 0.0006 | 13.6733 |
| ***CMPK2*** | cytidine/uridine monophosphate kinase 2 | 0.0299 | 0.0023 | 12.9929 |
| ***MMP9*** | matrix metallopeptidase 9 | 0.0572 | 0.0054 | 10.6879 |
| ***IFI44L*** | interferon induced protein 44 like | 0.0129 | 0.0012 | 10.5023 |

**Supplementary Table 3. Specific changes of EMT-related mRNA expression in microarray analyses.**

| **mRNA** | | **rate to β-actin** | | **rate to control** |
| --- | --- | --- | --- | --- |
| **Gene symbol** | **description** | **Platelet (+)** | **Control** |  |
| **Upregulated** | |  |  |  |
| ***MMP9*** | matrix metallopeptidase 9 | 0.0572 | 0.0054 | 10.6879 |
| ***ITGA5*** | integrin subunit alpha 5 | 0.01 | 0.0027 | 3.7166 |
| ***CTGF*** | connective tissue growth factor | 0.1699 | 0.0891 | 1.9069 |
| ***JAG1*** | jagged 1 | 0.0598 | 0.0322 | 1.8582 |
| ***FN1*** | fibronectin 1 | 1.9463 | 1.2011 | 1.6204 |
| ***NET1*** | neuroepithelial cell transforming 1 | 0.0533 | 0.0333 | 1.6 |
| ***SNAI2*** | snail family zinc finger 2 | 0.0918 | 0.0778 | 1.1802 |
| **Downregulated** | |  |  |  |
| ***CLDN1*** | claudin 1 | 0.0171 | 0.1094 | 0.1565 |
| ***KRT18*** | keratin 18, type I | 2.238 | 4.1833 | 0.535 |
| ***TJP1*** | tight junction protein 1 | 0.0251 | 0.0356 | 0.7062 |
| ***CDH1*** | cadherin 1 | 0.017 | 0.0222 | 0.769 |
| ***VIM*** | vimentin | 0.038 | 0.0471 | 0.8062 |
| ***SDC1*** | syndecan 1 | 0.0274 | 0.0326 | 0.8403 |

**EMT; epithelial–mesenchymal transition**

**Supplementary Table 4. Functional charts of Platelets-regulated genes in GO analysis (Top 100).**

| GO Term (Biological Process) | *p*-value | FDR |
| --- | --- | --- |
| GO:0071357~cellular response to type I interferon | 1.08E-11 | 2.13E-08 |
| GO:0060337~type I interferon signaling pathway | 1.08E-11 | 2.13E-08 |
| GO:0034340~response to type I interferon | 3.81E-11 | 7.53E-08 |
| GO:0051607~defense response to virus | 9.88E-11 | 1.95E-07 |
| GO:0034097~response to cytokine | 3.79E-10 | 7.48E-07 |
| GO:0016032~viral process | 8.39E-10 | 1.66E-06 |
| GO:0044764~multi-organism cellular process | 1.27E-09 | 2.50E-06 |
| GO:0048525~negative regulation of viral process | 2.29E-09 | 4.52E-06 |
| GO:1903901~negative regulation of viral life cycle | 4.41E-09 | 8.71E-06 |
| GO:0044419~interspecies interaction between organisms | 5.25E-09 | 1.04E-05 |
| GO:0044403~symbiosis, encompassing mutualism through parasitism | 5.25E-09 | 1.04E-05 |
| GO:0009615~response to virus | 7.98E-09 | 1.58E-05 |
| GO:0071345~cellular response to cytokine stimulus | 2.78E-08 | 5.49E-05 |
| GO:0019221~cytokine-mediated signaling pathway | 4.67E-08 | 9.22E-05 |
| GO:0045071~negative regulation of viral genome replication | 5.12E-08 | 1.01E-04 |
| GO:0043901~negative regulation of multi-organism process | 5.48E-07 | 0.001082271 |
| GO:0009605~response to external stimulus | 1.06E-06 | 0.002099234 |
| GO:0050792~regulation of viral process | 1.40E-06 | 0.002755562 |
| GO:0048519~negative regulation of biological process | 1.49E-06 | 0.002950966 |
| GO:0045069~regulation of viral genome replication | 2.31E-06 | 0.004564236 |
| GO:0006950~response to stress | 2.6346E-06 | 0.005203434 |
| GO:0009607~response to biotic stimulus | 2.64066E-06 | 0.005215403 |
| GO:0051707~response to other organism | 3.40173E-06 | 0.006718496 |
| GO:0043207~response to external biotic stimulus | 3.40173E-06 | 0.006718496 |
| GO:1903900~regulation of viral life cycle | 6.31126E-06 | 0.012464561 |
| GO:0006952~defense response | 9.92188E-06 | 0.019594775 |
| GO:0043903~regulation of symbiosis, encompassing mutualism through parasitism | 1.06082E-05 | 0.020950125 |
| GO:0002376~immune system process | 1.27624E-05 | 0.025203848 |
| GO:0048523~negative regulation of cellular process | 1.53735E-05 | 0.030359643 |
| GO:0098542~defense response to other organism | 3.08061E-05 | 0.060827315 |
| GO:0012501~programmed cell death | 4.26064E-05 | 0.084117761 |
| GO:0045087~innate immune response | 4.47767E-05 | 0.08840079 |
| GO:0006915~apoptotic process | 5.01833E-05 | 0.099069784 |
| GO:0043900~regulation of multi-organism process | 6.25403E-05 | 0.123450314 |
| GO:0051704~multi-organism process | 8.48887E-05 | 0.167529416 |
| GO:0035455~response to interferon-alpha | 0.000101888 | 0.201046326 |
| GO:0019079~viral genome replication | 0.000108986 | 0.215037057 |
| GO:0065007~biological regulation | 0.000149119 | 0.294111638 |
| GO:0001817~regulation of cytokine production | 0.000178304 | 0.351577959 |
| GO:0002252~immune effector process | 0.00018234 | 0.359523123 |
| GO:0070887~cellular response to chemical stimulus | 0.000184263 | 0.363308105 |
| GO:0008219~cell death | 0.000213948 | 0.421719847 |
| GO:0006955~immune response | 0.000217897 | 0.429488376 |
| GO:0010033~response to organic substance | 0.000254721 | 0.501897005 |
| GO:0048522~positive regulation of cellular process | 0.000293352 | 0.57780497 |
| GO:0050896~response to stimulus | 0.000319938 | 0.630014666 |
| GO:0042127~regulation of cell proliferation | 0.000324008 | 0.638003575 |
| GO:0001816~cytokine production | 0.000361358 | 0.711301136 |
| GO:0051179~localization | 0.000380559 | 0.748962432 |
| GO:0001819~positive regulation of cytokine production | 0.000422124 | 0.830441593 |
| GO:0051239~regulation of multicellular organismal process | 0.000427683 | 0.841332836 |
| GO:0044707~single-multicellular organism process | 0.000435562 | 0.856770044 |
| GO:0006810~transport | 0.000447439 | 0.880034608 |
| GO:0071310~cellular response to organic substance | 0.000463528 | 0.911542087 |
| GO:0050789~regulation of biological process | 0.000534375 | 1.050166736 |
| GO:0022607~cellular component assembly | 0.000616851 | 1.211314489 |
| GO:0032479~regulation of type I interferon production | 0.00065411 | 1.284032352 |
| GO:0007166~cell surface receptor signaling pathway | 0.000684646 | 1.343591339 |
| GO:0051240~positive regulation of multicellular organismal process | 0.000727618 | 1.427349719 |
| GO:0042493~response to drug | 0.000809275 | 1.586319852 |
| **GO:0008283~cell proliferation** | **0.000895432** | **1.753789951** |
| GO:0042221~response to chemical | 0.000901858 | 1.766269361 |
| GO:0044772~mitotic cell cycle phase transition | 0.000947653 | 1.855162969 |
| GO:0044699~single-organism process | 0.000993078 | 1.943262748 |
| GO:0071840~cellular component organization or biogenesis | 0.001012702 | 1.981299785 |
| GO:0044085~cellular component biogenesis | 0.001140008 | 2.227713824 |
| GO:0050794~regulation of cellular process | 0.001175438 | 2.296186994 |
| GO:0052547~regulation of peptidase activity | 0.001304865 | 2.545938531 |
| GO:0016043~cellular component organization | 0.001308437 | 2.552822683 |
| GO:0065003~macromolecular complex assembly | 0.001369351 | 2.670148089 |
| GO:0051234~establishment of localization | 0.001520858 | 2.961381727 |
| GO:0040011~locomotion | 0.002048804 | 3.969776362 |
| GO:0009892~negative regulation of metabolic process | 0.002116177 | 4.097742092 |
| GO:0044770~cell cycle phase transition | 0.002176826 | 4.212800113 |
| GO:0022610~biological adhesion | 0.002387717 | 4.611859086 |
| GO:0019043~establishment of viral latency | 0.002454505 | 4.73791031 |
| GO:0051259~protein oligomerization | 0.002460414 | 4.749054819 |
| GO:0060759~regulation of response to cytokine stimulus | 0.002723271 | 5.243562221 |
| **GO:0007155~cell adhesion** | **0.002803331** | **5.3936925** |
| GO:0051346~negative regulation of hydrolase activity | 0.002916926 | 5.606320382 |
| GO:0035456~response to interferon-beta | 0.00316705 | 6.072908449 |
| **GO:0016477~cell migration** | **0.00317802** | **6.093321811** |
| GO:0048518~positive regulation of biological process | 0.003218277 | 6.168198141 |
| GO:0032606~type I interferon production | 0.00324337 | 6.214839804 |
| GO:0031324~negative regulation of cellular metabolic process | 0.003388439 | 6.484064923 |
| GO:0014074~response to purine-containing compound | 0.003411489 | 6.526773731 |
| GO:0010628~positive regulation of gene expression | 0.003427629 | 6.556668258 |
| GO:0006928~movement of cell or subcellular component | 0.003493689 | 6.678930569 |
| GO:0042787~protein ubiquitination involved in ubiquitin-dependent protein catabolic process | 0.003655832 | 6.978379282 |
| GO:0097202~activation of cysteine-type endopeptidase activity | 0.003665215 | 6.995681459 |
| GO:0042993~positive regulation of transcription factor import into nucleus | 0.00373843 | 7.130572067 |
| GO:0050663~cytokine secretion | 0.003869088 | 7.37083572 |
| GO:0051716~cellular response to stimulus | 0.003881073 | 7.392845877 |
| GO:0030335~positive regulation of cell migration | 0.003890977 | 7.411029465 |
| GO:0010951~negative regulation of endopeptidase activity | 0.003907071 | 7.440571741 |
| GO:2000117~negative regulation of cysteine-type endopeptidase activity | 0.004067291 | 7.734179964 |
| GO:2000116~regulation of cysteine-type endopeptidase activity | 0.004110594 | 7.813383004 |
| GO:0031323~regulation of cellular metabolic process | 0.004204422 | 7.984776454 |
| GO:0009612~response to mechanical stimulus | 0.004248934 | 8.065977545 |
| GO:0052548~regulation of endopeptidase activity | 0.004285637 | 8.13288479 |

GO; gene ontology, FDR; false discovery rate.

**Supplementary Table 5. Functional pathways of Platelets-regulated genes in KEGG analysis.**

| KEGG Pathway Term | *p*-value | FDR |
| --- | --- | --- |
| hsa05168 Herpes simplex infection | 0.003 | 3.542 |
| hsa05162 Measles | 0.003 | 4.479 |
| hsa04512 ECM-receptor interaction | 0.005 | 6.759 |
| hsa05164 Influenza A | 0.007 | 8.331 |
| hsa04622 RIG-I-like receptor signaling pathway | 0.007 | 8.618 |
| hsa04623 Cytosolic DNA-sensing pathway | 0.01 | 12.489 |
| hsa05222 Small cell lung cancer | 0.011 | 13.924 |
| hsa04151 PI3K-Akt signaling pathway | 0.016 | 19.304 |

KEGG; Kyoto Encyclopedia of Genes and Genomes, FDR; false discovery rate.

**Supplementary Fig. 1 Novel methods for analysing effects of direct/indirect contact with platelets on malignant behaviour of GC cells.**

**
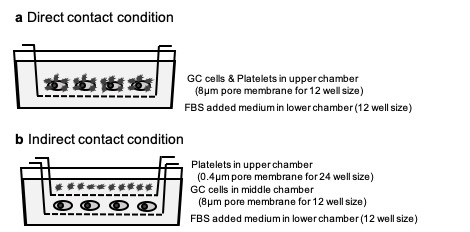
**

To evaluate effects of indirect contact with platelets on migration/invasion abilities of GC cells, we developed novel methods for cell function analyses. We used 0.4–µm-pore membrane for 24-well size for platelets, and 8–µm-pore membrane for 12-well size for GC cells. Using this system, migration/invasion assays were performed.

**Supplementary Fig. 2 Various conditions of (co-)incubations of GC cells (and platelets) for microarray analyses.**


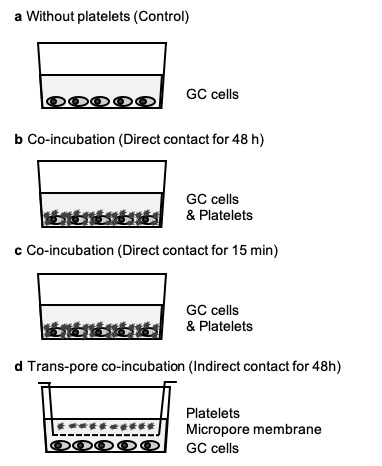


Microarray analyses were performed with RNAs derived from NUGC-3 cell lines, incubated in various conditions. **a** Incubated without platelets as control. **b** Co-incubated with platelets for 48 h. **c** Co-incubated with platelets for 15 min. **d** Co-incubated with platelets separated by 0.4 µm pore membranes for 48 h.

GC; gastric cancer.

**Supplementary Fig. 3 Relative expression levels of mRNAs.**


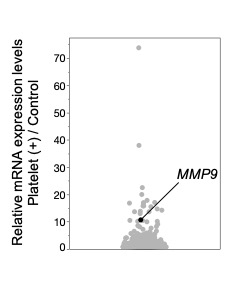


Each RNA expression level of GC cells co-incubated with platelets for 48 h was compared to those of control. All gray dots present each relative *mRNA* expression level, and the black point indicates that of *MMP9* (10.7 folds upregulated).

GC; gastric cancer, MMP9; matrix metalloproteinase 9.

**Detailed Methods**

*Patients and ethical concerns.*

In this study, all experimental procedures were performed using platelets derived from patients with advanced GC who were treated at the University of Yamanashi Hospital between November 2019 and April 2020. Blood samples were collected before therapeutic interventions, such as chemotherapy or surgical resection, and platelets were purified and subsequently used in various assays. The pathological findings of GC were defined according to the UICC classification (8th edition)^1^. This study was approved by the Ethic committee of the University of Yamanashi (approval number 2159) and was performed in accordance with the ethical standards of the Declaration of Helsinki and its later amendments^2^. Written informed consent for the use of samples and clinical data was obtained from all patients.

*Cell lines.*

The human GC cell lines NUGC-3 and MKN74 and the human normal mesothelial cell line Met-5A were used in this study. The GC cell lines were obtained from the Japanese Collection of Research Bioresources Cell Bank (Osaka, Japan), and Met-5a cells were purchased from ATCC (Manassas, VA, USA). GC cell lines were cultured in RPMI 1640 medium (Thermo Fisher Scientific, Inc., Waltham, MA, USA), and Met-5A cells were cultured in Medium 199 (Thermo Fisher Scientific, Inc.), both supplemented with 100 U/mL penicillin (Sigma-Aldrich; Merck KGaA, Darmstadt, Germany), 100 μg/mL streptomycin (Sigma-Aldrich; Merck KGaA), and 10% fatal bovine serum (Thermo Fisher Scientific, Inc.). The cells were grown in a 5% carbon dioxide atmosphere at 37°C.

*Platelets preparation.*

Platelets were purified from whole blood samples of patients with GC as previously reported^3,4^. In brief, 10 mL of anticoagulant whole blood was added to 1 mL of citrate solution, and platelet-rich plasma was obtained by centrifugation for 12 min at 1,000 rpm at room temperature. Then, platelets were purified and concentrated by washing with modified Tyrode buffer and centrifuging twice, using prostacyclin to prevent activation^4^. Platelets were subsequently used for various experiments.

*Electron microscopy of cancer cell-platelet contacts.*

NUGC-3 cells precultured for 24 h were co-cultured with platelets for 15 min and then harvested by EDTA treatment, washed with medium to eliminate free platelets, and collected by centrifugation. Cells used for scanning electron microscopy (SEM) imaging were fixed in phosphate-buffered 2% glutaraldehyde and post-fixed in 2% osmium tetra-oxide for 2 h in an ice bath. Then, the cells were dehydrated in a graded ethanol series and dried by t-Butyl alcohol freeze-drying. Dried cells were coated using an osmium plasma ion coater, and SEM was performed at 5kV using JSM-7500F (JEOL Ltd., Tokyo, Japan).

*Proliferation assay.*

GC cells were precultured for 24 h in 24-well plates at a concentration of 1.0 × 10^5^/mL, and then platelets were added to the wells; control wells did not have platelets. At 3 h, 24 h, and 48 h after co-culture, each well was washed with medium, fixed, and stained with Diff-Quick staining reagent (Sysmex, Kobe, Japan). Then, stained cancer cells, excluding platelets, were observed using the BZ-X710 All-in-One fluorescence microscope (Keyence Corp., Osaka, Japan) and counted using the BZ-X Analyzer Software (Keyence Corp.) in four independent fields at 100× magnification. Cell numbers at 24 h and 48 h were compared to those at 3 h after the start of co-culture. Each assay was performed in triplicate.

*Migration and invasion assays.*

The migration assay was performed using Falcon Cell Culture Inserts with 8-µm pore membranes (Corning, Corning, NY, USA). Biocoat Matrigel (BD Bioscience, Franklin Lakes, New Jersey, USA) was used in the invasion assay. In brief, 1 × 10^5^ cells were seeded in the upper chambers in FBS-free medium with or without platelets, and medium with 10% FBS was added in the lower chambers. After incubation for 24 h, cells that had not migrated or invaded through the pores were removed by cotton swabs. Then, migrated or invaded cells were fixed and stained with Diff-Quick staining reagent. Cells were counted in four independent fields at 100× magnification using the BZ-X710 All-in-One fluorescence microscope and BZ-X Analyzer Software. Each assay was performed in triplicate.

*Adhesion assay.*

The ability of GC cells co-incubated with platelets to adhere to mesothelial cells was evaluated by fluorescence microscopy (modified from previous reports^5,6^). The mesothelial Met-5A cells were grown to confluence on a 24-well plate. GC cells were detached from a dish by trypsin, washed with PBS, and probed with Hoechst (Thermo Fisher Scientific, Inc.), a nuclear counterstain for fluorescence microscopy, for 30 min at 37ºC. Then, the labelled cells were washed three times with PBS to remove free dye, and cells were divided evenly into two groups. Platelets in PBS were added to one group, and the same volume of PBS was added to the other group as a control. Both groups were incubated for 15 min at 37ºC. Then, labelled cells (5 × 10^4^ cells/well) were added to the mesothelial cells, and the plates were incubated for 60 min at 37ºC. After gentle washing to remove non-adherent cells, adherent cells were observed by fluorescence using the BZ-X710 All-in-One fluorescence microscope and counted using the BZ-X Analyzer Software in four independent fields at 100× magnification for each well. The adhesion abilities of GC cells co-incubated with platelets were compared to those of cells not co-incubated with platelets.

*Comparison between effects of direct and indirect contact with platelets on malignant behaviours of GC cells.*

Cell function assays involving indirect contact between GC cells and platelets were performed. In proliferation and adhesion assays, Falcon Cell Culture Inserts with 0.4-µm pore membranes (Corning) were used to create conditions for indirect contact. Other parameters, such as cell and platelet quantities and contact time, were identical to those of direct contact. On the other hand, migration and invasion assays were performed using a novel method developed in this study. Two chambers with different sizes were used. For creating the condition for indirect contact with platelets, 6.5 mm Transwell® with 0.4-µm Pore Membrane Insert (Product Number 3470, Corning) was used as the upper chamber. Subsequently, for evaluation of migration/invasion abilities of GC cells, Falcon® Permeable Support for 12-well Plate with 8.0 µm Transparent PET Membrane (Product Number 353182, Corning) was used. In addition, for invasion assay, these 12-well chambers were precoated with Corning® Matrigel® Basement Membrane Matrix (Corning) as per the manufacturer’s instructions. These two chambers were cumulated and set on 12-well plates, and the assays were performed. Other conditions and procedures were the same as in the rest of the study.

*RNA extraction and microarray analysis.*

NUGC-3 cells were co-cultured with platelets in 6-well plates for 15 min or 48 h. Cells were also co-cultured with platelets separated by 0.4-µm pore membranes for 48 h. Cells cultured for 48 h without platelets were used as a control (Supplemental Fig. 1). Total RNA was extracted using TRIzol Reagent (Thermo Fisher Scientific, Inc.) and RNeasy Mini Kit (Qiagen, Hilden, Germany). The RNA concentration was measured using a NanoDrop 2000/2000c spectrophotometer (Thermo Fisher Scientific, Inc.). Microarray analysis was performed to identify changes in mRNA expression after co-culture with platelets. Briefly, Amino Allyl Message Amp II RNA Amplification Kit (Ambion, Tokyo, Japan) was used to synthesize Amino Allyl RNA. CyDye coupling and fragmentation were performed following the protocol supplied by the manufacturer (Toray Industries Inc., Tokyo, Japan). RNA samples were hybridized to Toray 3D-Gene Human 25k chips (24460 probes) for 16 h at 37°C by shaking. A 3D-Gene Scanner 3000 (TORAY) was used for scanning microarrays. After subtracting the background signal, gene expression levels were normalized against the β-actin levels in each sample.

Following microarray analysis, mRNAs with more than 1.5-fold differences were considered significantly changed and were selected for further analyses. Enrichment analyses of the altered mRNAs and functional pathways were performed using GO enrichment analysis and KEGG pathway analysis, respectively. These results were analysed using DAVID Bioinformatics Resources 6.8 software (Laboratory of Human Retrovirology and Immunoinformatics, Frederick, Maryland, USA).

*Quantitative reverse transcription-polymerase chain reaction.*

cDNA was synthesized from extracted RNA with the High-Capacity cDNA Reverse Transcription Kit (Thermo Fisher Scientific, Inc.) following the manufacturer’s protocols. Subsequently, quantitative reverse transcription-polymerase chain reaction (qRT-PCR) was performed using 1 μL of cDNA, 5 μL of Power SYBR Green Master Mix (Thermo Fisher Scientific, Inc.), 1 μL of primers (FASMAC Co., Ltd., Kanagawa, Japan), and 3 μL of nuclease-free water. The qRT-PCR conditions were as follows: preheat for 10 min at 95°C and 40 cycles at 95°C for 15 sec and at 60°C for 60 sec. The qRT-PCR reactions were performed on a 7500 Real-Time PCR machine (Applied Biosystems) and analysed using the 7500 System SDS software. The RNA expression levels were normalized to the expression level of β-actin and analysed using the ΔΔCq method. The sequences of the primers were as follows; *ACTB* (forward, 5’-CACCATT-GGCAATGAGCGGTTC-3’ and reverse, 5’-AGGTCTTTGCGGATGTCCACGT-3’), *MMP9* (forward, 5’-CCAACTACGACCGGGACAAG-3’ and reverse, 5’-AAGTGAA-GGGGAAGACGCAC-3’).

*Western blotting.*

GC cells co-incubated with platelets from patients with AGC or control cells were harvested in RIPA buffer (Thermo Fisher Scientific, Inc.) supplemented with protease inhibitors (Cell Signaling Technology, Inc., Danvers, MA, USA). The protein concentration was measured using a NanoDrop 2000/2000c spectrophotometer. Cell lysates were separated by 10% sodium dodecyl sulphate-polyacrylamide gel electrophoresis at a concentration of 40 µg/lane and then transferred onto polyvinylidene fluoride (PVDF) membranes (Merck USA, Minneapolis, MN, USA). The PVDF membranes were incubated in Odyssey blocking buffer (LI-COR Bioscience, Lincoln, NE, USA) and PBS for 1 h at room temperature. The PVDF membranes were incubated with primary antibodies overnight at 4ºC. Anti-MMP9 (dilution 1:2000) and anti-β-actin (dilution 1:1000) antibodies were purchased from Abcam (Cambridge, MA, USA). Then, the membranes were washed four times for 5 min in PBS and incubated with goat anti-rabbit IgG conjugated to horseradish peroxidase (dilution 1:2000, Abcam) for 1 h at room temperature. The membranes were probed by an ECL Prime Western Blotting Detection reagent (Thermo Fisher Scientific, Inc.). Chemiluminescence imaging was performed using Image Quant LAS 4000 (GE Healthcare Life Sciences, Buckinghamshire, England).

*Statistics.*

All quantitative values were represented as mean ± standard error or median value and statistically analysed using the Student’s t-test. *P* < 0.05 was considered to indicate a statistically significant result. All statistical analyses were conducted with EZR (Saitama Medical Center, Jichi Medical University, Saitama, Japan), which is a graphical user interface for R (The R Foundation for Statistical Computing, Vienna, Austria)^7^. Graphs were prepared using EZR and JMP 13 (SAS Institute Inc., Cary, NC, USA).

**Detailed Method: References**

1. UICC. *TNM classification of malignant tumours*, 8th edn. (John Wiley & Sons, Ltd: New York, USA, 2017)

2. World Medical Association Declaration of Helsinki: ethical principles for medical research involving human subjects. *Jama* **310**, 2191-2194 (2013).

3. Satoh, K., Fukasawa, I., Kanemaru, K., Yoda, S., Kimura, Y., Inoue, O. *et al.* Platelet aggregometry in the presence of PGE(1) provides a reliable method for cilostazol monitoring. *Thromb Res* **130**, 616-621 (2012).

4. Suzuki-Inoue, K., Inoue, O., Frampton, J. & Watson, S. P. Murine GPVI stimulates weak integrin activation in PLCgamma2-/- platelets: involvement of PLCgamma1 and PI3-kinase. *Blood* **102**, 1367-1373 (2003).

5. Ranieri, D., Raffa, S., Parente, A., Rossi Del Monte, S., Ziparo, V. & Torrisi, M. R. High adhesion of tumor cells to mesothelial monolayers derived from peritoneal wash of disseminated gastrointestinal cancers. *PLoS One* **8**, e57659 (2013).

6. Kimura, R., Yoneshige, A., Hagiyama, M., Otani, T., Inoue, T., Shiraishi, N. *et al.* Expression of cell adhesion molecule 1 in gastric neck and base glandular cells: Possible involvement in peritoneal dissemination of signet ring cells. *Life Sci* **213**, 206-213 (2018).

7. Kanda, Y. Investigation of the freely available easy-to-use software 'EZR' for medical statistics. *Bone Marrow Transplant* **48**, 452-458 (2013).
